# ATF4‐mediated stress response as a therapeutic vulnerability in chordoma

**DOI:** 10.1002/1878-0261.70176

**Published:** 2025-11-29

**Authors:** Lucia Cottone, James Dunford, Eleanor Calcutt, Vicki Gamble, Filiz Senbabaoglu Aksu, Lorena Ligammari, Georgina Wherry, Giorgia Gaeta, John C. Christianson, Adrienne M. Flanagan, Udo Oppermann, Adam P. Cribbs

**Affiliations:** ^1^ Department of Pathology University College London Cancer Institute UK; ^2^ Botnar Research Centre, Nuffield Department of Orthopaedics, Rheumatology and Musculoskeletal Sciences, National Institute of Health Research Oxford Biomedical Research Unit (BRU) University of Oxford UK; ^3^ Department of Musculoskeletal Radiology Royal National Orthopaedic Hospital Stanmore UK; ^4^ Oxford Centre for Translational Myeloma Research University of Oxford UK

**Keywords:** ATF4, chordoma, prolyl‐tRNA synthetase, stress response

## Abstract

Chordoma, a rare primary bone malignancy, currently lacks effective targeted therapies. Despite surgical resection and adjuvant radiotherapy, prognosis remains poor. Recent preclinical studies have highlighted potential therapeutic targets, including the transcription factor T‐box transcription factor T (TBXT). However, clinical outcomes associated with therapies targeting TBXT remain underexplored or have been modest, warranting further investigation. In this study, we investigated the therapeutic potential of transfer RNA (tRNA) synthetase inhibitors in chordoma treatment. Focused compound screening identified distinct chemotypes targeting human glutamyl‐prolyl‐tRNA synthetase (EPRS) as being effective in reducing cell viability in chordoma cell lines through a cyclic AMP‐dependent transcription factor (ATF4)‐mediated stress response rather than through TBXT regulation. Mechanistically significant upregulation of *ATF4* and associated stress response genes was identified with consecutive pro‐apoptotic DNA damage‐inducible transcript 3 protein (DDIT3)‐mediated cell death. The prototypic EPRS inhibitor halofuginone demonstrated significant tumour growth inhibition in an *in vivo* patient‐derived xenograft model. These results suggest that targeting metabolic stress pathways via ATF4 activation presents a novel therapeutic approach for chordoma, warranting further clinical investigation.

## Graphical abstract

We screened 5 chordoma cell lines against 100+ inhibitors of epigenetic and metabolic pathways and kinases and identified halofuginone, a tRNA synthetase inhibitor. Mechanistically halofuginone induces an integrated stress response, with eIF2alpha phosphorylation, activation of ATF4 and its target genes CHOP, ASNS, INHBE leading to cell death. Halofuginone significantly reduced tumour growth in a chordoma PDX model.

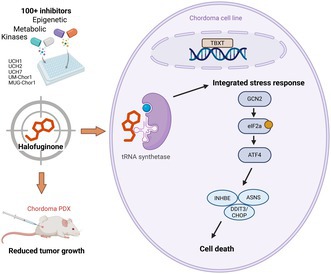

**Abbreviations**
AAALAC: Association for Assessment and Accreditation of Laboratory Animal Care
AMP: adenosine monophosphate
ANOVA: analysis of variance
ASNS: asparagine synthetase [glutamine‐hydrolysing]
ATF4: activating transcription factor 4
BSA: bovine serum albumin
cDNA: complementary deoxyribonucleic acid
CDC20B: cell division cycle 20 homologue B
CDK4/6: cyclin‐dependent kinase 4/6
CHOP (DDIT3): C/EBP homologous protein/DNA damage‐inducible transcript 3
DAB: diaminobenzidine
DESeq2: differential expression sequencing 2
DMSO: dimethyl sulfoxide
DNA: deoxyribonucleic acid
DPX: distyrene plasticizer xylene
EPRS: glutamyl‐prolyl‐tRNA synthetase (GluProRS)
ER: endoplasmic reticulum
FBS: fetal bovine serum
FFPE: formalin‐fixed paraffin‐embedded
GCN2: general control nonderepressible 2
GO: gene ontology
HEPA: high‐efficiency particulate air
HRP: horseradish peroxidase
IACUC: Institutional Animal Care and Use Committee
IC50: half maximal inhibitory concentration
IHC: immunohistochemistry
IMDM: Iscove's modified Dulbecco's medium
INHBE: inhibin beta E chain
ISR: integrated stress response
ISRIB: integrated stress response inhibitor
KDM: lysine demethylase
KEGG: kyoto encyclopedia of genes and genomes
PBS: phosphate‐buffered saline
PCR: polymerase chain reaction
PDX: patient‐derived xenograft
PERK: protein kinase RNA‐like endoplasmic reticulum kinase
PI: propidium iodide
ProRS: prolyl‐tRNA synthetase
qPCR: quantitative polymerase chain reaction
RNA: ribonucleic acid
RNA‐seq: RNA sequencing
RRID: research resource identifier
RT: room temperature
SARS‐CoV‐2: severe acute respiratory syndrome coronavirus 2
SD: standard deviation
SQ: subcutaneous
STR: short tandem repeat
TBXT: T‐Box transcription factor T
TBS: tris‐buffered saline
tRNA: transfer ribonucleic acid

## 1 Introduction

Chordoma is an uncommon malignancy presenting primarily along the spinal axis, exhibiting notochordal differentiation and thought to originate from primitive notochordal remnants [1]. This rare primary bone cancer remains challenging to treat, with the median survival rate for affected patients being approximately 7 years [2, 3]. The current standard of care for chordoma involves surgical resection complemented by adjuvant proton or photon radiotherapy. Despite these interventions, the prognosis remains poor.

There is currently no targeted therapy approved for chordoma; however, recent preclinical studies have identified several novel therapeutic targets [4]. The efficacy of tyrosine kinase inhibitors has been explored [5, 6] and although these studies have demonstrated some benefit, the clinical outcomes have been modest (Clinical trial NCT03083678), and there has been limited improvement in patient survival rates. The role of immunotherapy in managing chordoma is an active area of investigation, with early studies suggesting possible therapeutic benefits [4].

The transcription factor TBXT, essential for notochord development, is epigenetically silenced in the human foetus at approximately 12 weeks of gestation, leading to the regression of the notochord [7]. TBXT is expressed in virtually all chordomas, serving as both a diagnostic marker and a potential therapeutic target [1, 8]. Evidence suggests that post‐transcriptional modifications of histones also regulate TBXT expression [9], indicating the potential of epigenetic inhibitors as a promising therapeutic strategy to selectively target TBXT.

In a previous study, we employed a targeted screen of epigenetic‐modifying compounds on chordoma cell lines, demonstrating that inhibitors of lysine demethylases (KDM) effectively induce cell death *in vitro* by diminishing TBXT expression [9]. However, these compounds exhibit suboptimal pharmacodynamics, rendering them unsuitable for clinical application, and highlighting the need for further molecular screening.

Given its central role in regulating cellular adaptation to various metabolic stress, the integrated stress response (ISR) has emerged as a key modulator of cancer cell survival [10]. The ISR is initiated by stress‐sensing kinases, including eIF‐2‐alpha kinase (GCN2), which responds to amino acid deprivation and eukaryotic translation initiation factor 2‐alpha kinase 3 (PERK), which is activated by endoplasmic reticulum (ER) stress, and converges on the phosphorylation of the eukaryotic translation initiation factor 2 subunit 1 (eIF2α). ISR results in global translational attenuation but enables the selective translation of stress‐responsive transcription factors such as cyclic AMP‐dependent transcription factor (ATF4) and DNA damage‐inducible transcript 3 protein (DDIT3)/C/EBP homologous protein (CHOP) and, depending on the context, promotes adaptive responses or apoptotic outcomes [11].

In this study, we report the findings from an enhanced and broadened drug screening effort for chordoma, incorporating additional epigenetic inhibitors and small molecules, aimed at identifying novel therapeutic targets for this malignancy. We have identified two promising compounds that exhibit potential for chordoma treatment, and we elucidate their mechanisms of action, which involve triggering an ATF‐driven stress response in both *in vitro* and *in vivo* models.

## 2 Materials and methods

### 2.1 Cell lines

Cell lines UCH‐1 (RRID : CVCL_4988), UCH‐2 (RRID : CVCL_4989), UCH‐7 (RRID : CVCL_IR23), UM‐Chor1 (RRID : CVCL_1D68) and MUG‐Chor (RRID : CVCL_9277) were cultured as previously described [12]. Cell lines were either obtained from our collaborators in University Hospitals of Ulm, Germany (U‐CH1, U‐CH2, MUG‐Chor1) or from the Chordoma Foundation (UM‐Chor1, www.chordomafoundation.org) or developed at UCL (UCH‐7) [12]. All chordoma cell lines derive from sacral tumours except UM‐Chor1, which was generated from a clival chordoma. Briefly, cells were cultured in 4 : 1 Iscove's modified Dulbecco's medium (IMDM) (Gibco/Thermo Fisher Scientific, NY, USA): RPMI 1640 (Gibco/Thermo Fisher Scientific) with 10% fetal bovine serum (FBS) (Cat. F9665, Sigma‐Aldrich, St Louis, MO, USA) and 1% Penicillin and Streptomycin (cat. 15070063; Life Technologies/Thermo Fisher Scientific). Cell lines have been authenticated in the past 3 years by short tandem repeat (STR) analysis and were regularly tested for cross‐contaminating. Cells were also regularly confirmed to be mycoplasma‐free using the EZ‐PCR Mycoplasma Test Kit (K1‐0210; Geneflow, Lichfield, Staffordshire, UK).

### 2.2 Compound screening library

Drug screening was conducted as previously described [9]. Briefly, human chordoma cell lines (U‐CH1, U‐CH2, U‐CH7, MUG‐Chor and UM‐Chor1) were seeded at 5000 cells per well in 96‐well plates and incubated overnight. Cells were treated with small molecule inhibitors or vehicle control (0.1% DMSO) for three to 6 days (see Tables S1 and S2 for compound details). Cell viability was assessed using Presto Blue Cell Viability Reagent (Thermo Fisher Scientific) and normalized to vehicle controls. Each assay was performed in triplicate, with three independent repetitions. IC50 values were determined using a seven‐point dose–response curve, starting at 50 μmol·L^−1^, and calculated using Prism version 10.

For RNAseq, western blot and individual assays cells were treated with halofuginone or haloguginol at 70 nm, for the time indicated.

### 2.3 Proliferation monitoring assay

Cells were seeded in 96‐well plates at a density of 5000 cells per well in 100 μL of complete culture medium and left to adhere overnight. Prior to seeding, cell viability was assessed using trypan blue exclusion, and only cultures with viability > 90% were used. Cell imaging was performed using the Celigo (Revvity, MA, USA), an automated high‐content imaging system. The instrument was calibrated according to the manufacturer's guidelines. Cells were labelled with Hoechst and propidium iodide (PI) staining. Hoechst stains all cells (both live and dead), while PI only stains dead cells with compromised membranes, allowing for the differentiation between live and dead cells. Plates were placed in the Celigo, and brightfield images were captured using a 4× objective lens. Images were acquired at multiple time points (0, 24, 48 and 72 h) to monitor cell proliferation and morphology changes. Images were analysed using Celigo's integrated software, which utilized automated analysis algorithms to obtain cell counts and confluency measurements by detecting cells based on optimized size, shape and intensity thresholds for each cell line. The cell counting module quantified the number of cells per well, excluding debris and dead cells through appropriate size and circularity filters. Confluency was measured as the percentage of well area occupied by cells, with adjusted thresholds ensuring accurate cell boundary segmentation. Cell proliferation rates were determined by normalizing cell counts at subsequent time points to the initial count, generating growth curves and calculating doubling times using exponential growth models.

### 2.4 RNA sequencing of chordoma cell lines

Total RNA was extracted from cell lines and tumour samples from treated mice (see below) using TRIzol reagent, followed by purification with the Direct‐zol RNA Miniprep Kit (Zymo Research, Irvine, CA, USA), which includes an on‐column DNase I digestion step to remove genomic DNA. Poly(A) RNA was isolated from the total RNA using the NEBNext Poly(A) mRNA Magnetic Isolation Module (New England Biolabs, MA, USA). First‐strand cDNA libraries were prepared using the NEBNext Ultra Directional RNA Library Prep Kit (New England Biolabs) and sequenced in a 41 bp paired‐end configuration on a NextSeq 500 platform (Illumina, CA, USA).

### 2.5 Illumina sequencing data analysis

Sequencing reads were pseudoaligned to the hg38 reference transcriptome using Kallisto [13]. The quality of the pseudoalignment was assessed with FastQC. Differential gene expression analysis was conducted using the DESeq2 package [14], with genes considered differentially expressed if the adjusted *P* value was < 0.01. Heatmaps of differentially expressed genes were generated using the pheatmap and ggplot2 packages in R.

### 2.6 Nanopore sequencing

Library preparation was conducted using the cDNA‐PCR Barcoding Kit V14 (SQK‐PCB114.24; Oxford Nanopore Technologies, Oxford, UK) according to the manufacturer's protocol. Briefly, the amplified cDNA was end‐repaired and dA‐tailed using the NEBNext End Repair/dA‐Tailing Module (New England Biolabs). The barcoded adapters provided in the kit were then ligated to the prepared cDNA. The barcoded cDNA was purified using AMPure XP beads (Beckman Coulter, CA, USA) to remove excess adapters and enzymes. The concentration and quality of the barcoded libraries were assessed using the Qubit 4 Fluorometer (Thermo Fisher Scientific) and the Agilent 2100 Bioanalyzer. The samples were then pooled and the library was loaded onto a PromethION 24 device (Oxford Nanopore Technologies) using R10.4.1 flow cells. Sequencing was initiated and run according to the manufacturer's instructions. Real‐time base calling was performed using MinKNOW software (Oxford Nanopore Technologies).

### 2.7 Nanopore sequencing data analysis

Raw sequencing data were processed using Guppy basecaller (v6.5.7; Oxford Nanopore Technologies) using the high accuracy settings to generate high‐quality reads. Subsequent data analysis, including alignment, transcript quantification and differential expression analysis, was performed using Minimap2 [15] for alignment and DESeq2 for differential expression analysis. Quality control of the sequencing data was assessed using FastQC.

### 2.8 Western blot and qPCR

Western blot and band intensity quantification was performed as described previously [16]. Antibodies are listed in Table S3. All antibodies were blocked in 5% bovine serum albumin (BSA; Sigma‐Aldrich) and made up in TBS, with 0.01% Tween and 5% BSA. qPCR was performed as described previously [9] using primers reported in Table S4.

### 2.9 Animal experiments

Animal experiments were performed at South Texas Accelerated Research Therapeutics (START) under the following IACUC#: START #09‐001. The antitumor activity of halofuginone and palbociclib was tested in a patient‐derived xenograft (PDX) model, designated SF8894 [17], in 6–12‐week‐old female Athymic Nude (Crl : NU(NCr)‐Foxn1nu) immune‐deficient mice (Charles River Laboratories, Wilmington, MA, USA). Data collected from this study included animal weights, observations and tumour dimensions. This information was used to determine agent tolerability based on weight change and gross physiologic changes and anticancer activity based on tumour growth inhibition or regression with the data analysis endpoint at Day 42.

Mice weighing an approximate minimum of 20 grams on Day 0, are acclimated for a minimum of 24 h and housed on irradiated corncob bedding (Teklad) in individual HEPA ventilated cages (Sealsafe® Plus) (Techniplast, PA, USA) with irradiated corncob bedding, nesting sheets and plastic housing, on a 12‐h light–dark cycle at 70–74 °F (21–23°C) and 40–60% humidity. Animals are fed water *ad libitum* (reverse osmosis, 2 ppm Cl_2_) and an irradiated standard rodent diet (Teklad 2919) consisting of 19% protein, 9% fat and 4% fibre. Animals were identified via ear notch.

The SF8894 is a PDX model from an adult patient with skull‐base conventional chordoma. Tumour fragments were harvested from host animals and implanted SQ in the right flank into immune‐deficient mice, allowed to grow for approximately 20 days and the study initiated at a mean tumour volume of approximately 125–250 mm^3^ upon which the tumour‐bearing animals were randomized into the different groups. Animals bearing xenografts were excluded if they did not reach sufficient size. Animals were dosed daily with halofuginone (S8144, Selleckchem, 1 mg·kg^−1^ in 10% DMSO, 90% Sterile Saline), Palbociclib (Lot PBC‐106, LC Labs, 75 mg·kg^−1^ in 50 mm Sodium Lactate, pH4) or no treatment as control. Administered volume: 10 mL·kg^−1^. Tumour measurements were performed twice weekly by calliper and no ulcerations were allowed. The tumour endpoint in this experiment was 1000 mm^3^ or 42 days. Data capture was direct electronic with digital scale or digital calliper, and data were stored in Redundant Cloud Server. Toxicity was assessed by weight change or changes in eating/feeding or mobility. Mice were weighed twice weekly throughout the course of the drug treatment.

Tumour tissue was collected at termination of the study after the animals were euthanized. For freezing and storage in liquid nitrogen, a tumour fragment no greater than 9 × 9 mm was placed in a flash freeze vial on ice and transferred to liquid nitrogen for at least 30 min and then placed at −80°C for long‐term storage. For FFPE, a 5 × 5 mm core fragment was obtained while on ice (avoiding tumour ends and rim) and placed in 10% formalin fixation. Samples were sent in formalin for paraffin embedding 48 h after collection. Experiments were carried out in accordance with the ARRIVE 2.0 guidelines.

### 2.10 Immunohistochemistry for evaluating Ki‐67 and cleaved Caspase‐3 expression

Ki‐67 and cleaved Caspase‐3 expression were evaluated using immunohistochemistry (IHC). Tissue sections (5 mm) were cut from formalin‐fixed, paraffin‐embedded (FFPE) tissue blocks. For Ki‐67, deparaffinization and antigen retrieval were performed using EnVision FLEX Target Retrieval Solution, high pH (DAKO; Agilent Technologies, Santa Clara, CA, USA) in a PT Link pre‐treatment module (DAKO; Agilent). Slides were incubated at room temperature with Ready Probes Endogenous HRP and AP Blocking Solution (Invitrogen) for 15 min to block endogenous peroxidase activity. Slides were incubated for a further 20 min at room temperature with phosphate‐buffered saline (PBS) solution containing 10% normal goat serum (Abcam, Cambridge, UK) and 3% bovine BSA to block non‐specific protein binding. Slides were then incubated with Ki‐67 antibody (27 309‐1‐AP; Polyclonal, ProteinTech, IL, USA) for 30 min at room temperature and washed in PBS. Ki‐67 signal was detected with diaminobenzidine (DAB) substrate provided with the Mouse and Rabbit Specific HRP/DAB IHC Detection Kit—Micropolymer (Abcam) according to the manufacturer's instructions. Slides were counterstained with haematoxylin (Sigma‐Aldrich). For cleaved Caspase‐3 expression, slides were stained using Leica BOND‐MAX, with antigen retrieval ER1 for 10 min and incubated with Cleaved Caspase‐3 (Asp175) Antibody (Cat 9661, Cell Signalling Technologies) at a dilution of 1 : 500. Slides were sequentially dehydrated with increasing concentrations of ethanol and xylene and mounted using DPX mounting medium (Fisher Scientific). Whole‐slide images were acquired with Motic EasyScan slide scanner (Motic, Richmond, BC, Canada) and Leica Aperio GT 450 DX slide scanner.

Digital image analysis of immuno‐labelled IHC whole‐slide images was performed using the open‐source image analysis program QuPath version 0.5.1 [18]. Each immune‐labelled section was reviewed to identify and annotate the area of greatest cleaved Caspase3 or Ki67 positivity, that is, the proliferative hotspot. Scans were imported into QuPath and cell detection was performed. Areas of necrosis, tissue folds and artefacts were excluded from analysis, as previously described [18, 19].

### 2.11 Statistics

Data were analysed using graphpad prism (version 10). Statistical analyses included one‐way ANOVA followed by *post hoc* Tukey's test for multiple comparisons. Results were expressed as mean ± standard deviation (SD) from a minimum of three independent experiments. A *P* value < 0.05 was considered statistically significant. For animal model data, day 0 tumour volumes were analysed with either ANOVA followed by Dunnett's test for three or more groups, or Student's *t*‐test for comparisons involving fewer than three groups.

## 3 Results

### 3.1 tRNA synthetase inhibitors can induce cell apoptosis in chordoma cell lines

**Fig. 1:**
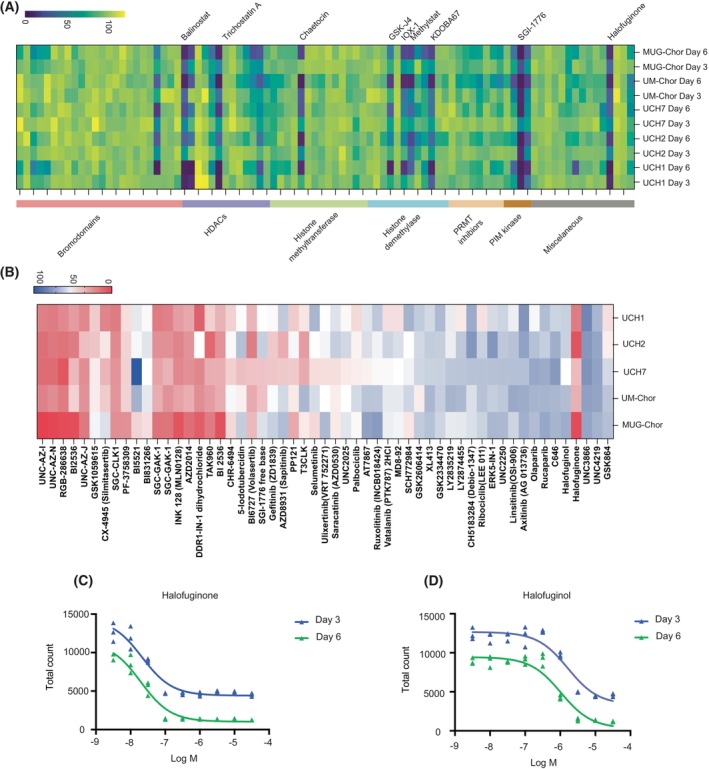
Identification of halofuginone as an effective agent in chordoma through drug screening targeting metabolic pathways, kinases and novel epigenetic inhibitors. (A, B) Heatmaps illustrating the screening of 229 small‐molecule probes targeting enzymes involved in metabolic pathways, epigenetic modifications (A) and kinases (B) across five chordoma cell lines. Each row corresponds to the mean expression of three replicates, while each column represents an individual compound, grouped by inhibitor class. The data are presented as fractional viability relative to the vehicle (DMSO) control. *N* = 3 replicates per condition. (C, D) Dose–response curves for halofuginone (C) and halofuginol (D) treatment in the UM‐Chor1 chordoma cell line over 3 and 6 days. Each point denotes the mean of three independent experiments. *N* = 3 replicates per condition.

We previously conducted a comprehensive screening of a library of tool compounds targeting chromatin proteins, including epigenetic readers, writers and erasers, in chordoma cell lines [9]. In our current study, we expanded these screens to encompass compounds targeting metabolic pathways, kinases and novel epigenetic inhibitors. MUG‐Chor, UM‐Chor1, UCH7, UCH2 and UCH1 chordoma cell lines were treated for three and 6 days in the presence of the compounds and then cell viability was assessed (Fig. 1A,B and Fig. S1, Fig. S2 and Tables S1, S2). Our findings confirm previous results, demonstrating that histone deacetylase inhibitors, demethylase inhibitors and PIM kinases are effective in killing three chordoma cell lines (UCH‐1, MUG‐Chor, and UM‐Chor1). Specifically, we reaffirmed dependencies on H3K27me3 demethylase (KDOBA67 [9]) and HDAC targets (Belinostat [20]).

Both screening libraries included the tRNA synthetase inhibitor halofuginone, which reduced cell viability across all cell lines (Fig. 1A). Halofuginone, a febrifugine quinazoline alkaloid derivative, inhibits the ProRS activity of GluProRS (EPRS) in a proline‐competitive manner [21]. Additionally, the library included halofuginol, a tRNA synthetase inhibitor with reduced potency. Our results show that both halofuginone and halofuginol effectively induce cell death in chordoma cell lines, with EC50 values in the low micromolar range (Figs 1C,D and S1). EPRS inhibitors have also shown promise in treating multiple myeloma [22, 23] and SARS‐CoV‐2 [24] infection.

### 3.2 Convergent transcriptional reprogramming by halofuginone and halofuginol in chordoma

**Fig. 2:**
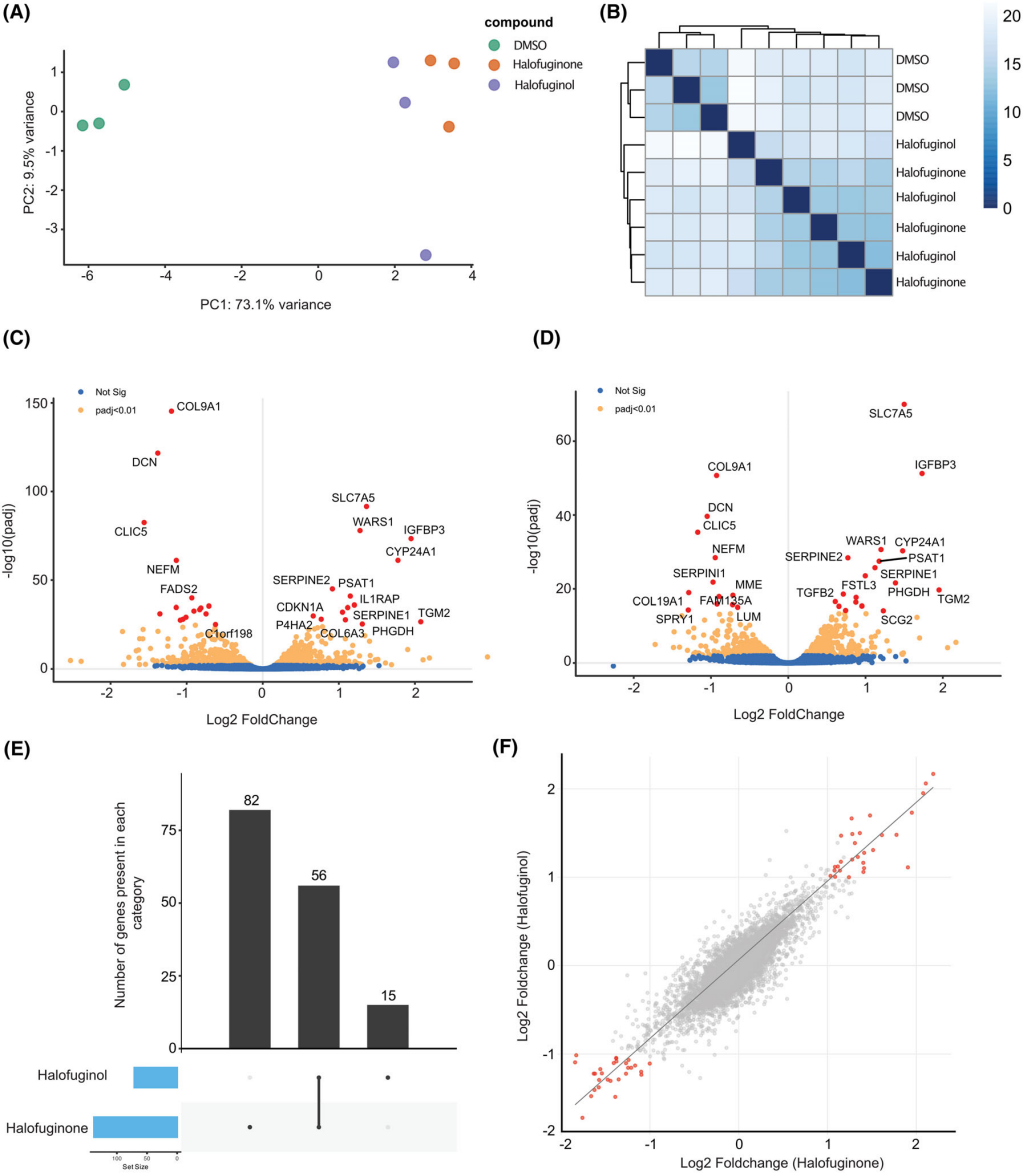
Transcriptomic profiling of chordoma cells in response to halofuginone and halofuginol. (A) Principal component analysis (PCA) of MUG‐chor cells treated with DMSO, halofuginone or halofuginol for 3 days. Each dot represents a biological replicate (*n* = 3 replicates per condition), demonstrating the variance in transcriptomic profiles across treatments. (B) Heatmap of pairwise Euclidean distances among transcriptomic profiles of MUG‐chor cells treated with DMSO, halofuginone or halofuginol for 3 days, illustrating the degree of similarity or dissimilarity between conditions. Each point represents a biological replicate (*n* = 3 replicates per condition). (C, D) Volcano plots depicting differential gene expression in MUG‐chor cells treated with halofuginone (C) or halofuginol (D) for 3 days. Genes with an adjusted *P* value (Padj) < 0.05 are highlighted in orange, while non‐significant genes are shown in blue, genes with an adjusted *P* value (Padj) < 0.05 and fold change > 2 are shown in red. Data represent the average of 3 biological replicates per condition. (E). Bar plot illustrating the number of differentially regulated genes across treatment conditions: halofuginone alone (left bar), jointly regulated by both halofuginone and halofuginol (middle bar), and halofuginol alone (right bar). *n* = 3 biological replicates per condition. (F) Scatter plot displaying the correlation of Log_2_ fold changes in gene expression between halofuginone and halofuginol treatments, with each point representing an individual gene. Genes with an adjusted *P* value (Padj) < 0.05 and Fold Change > 2 are shown in red. *n* = 3 biological replicates per condition.

To investigate the transcriptional changes induced by halofuginone and halofuginol treatments, given their potential to induce apoptosis in chordoma cell lines, we performed RNA sequencing on MUG‐Chor cell lines treated with these compounds for 3 days at their EC50 concentrations (Fig. 2 and Table S5). Principal component analysis (PCA) of the gene expression data revealed a clear separation between the treated and control groups, with halofuginone and halofuginol treatments clustering closely together, indicating similar transcriptional profiles (Fig. 2A). This similarity is further supported by the significant overlap in differentially expressed genes between the two treatments (Fig. 2B–D). Specifically, 56 genes were commonly regulated, and the log_2_ fold changes in gene expression showed a strong correlation, underscoring the overlapping effects of these compounds (Fig. 2E,F). Despite these significant gene expression changes, GO and KEGG pathway analyses did not reveal notable pathway enrichments. However, a consistent downregulation of *cell division cycle protein 20 homologue B* (*CDC20B*) was observed in both datasets, suggesting a role in cell cycle regulation.

### 3.3 Early response genes to tRNA synthetase inhibitors suggest a stress‐induced mechanism of action

**Fig. 3:**
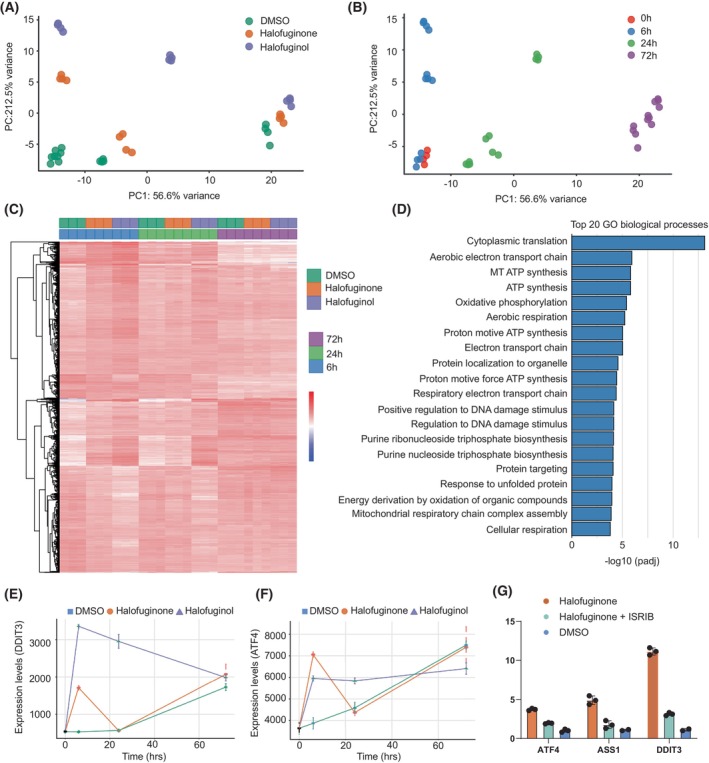
Transcriptomic analysis of early and late gene expression reveals tRNA synthetase inhibitors induce a stress‐related mechanism of action. (A, B) Principal Component Analysis (PCA) of MUG‐chor cells treated with DMSO, halofuginone or halofuginol at the indicated time points of 0 h, 6 h, 24 h and 72 h after treatment. PCA plots are clustered by treatment (A) or by time point (B). Each dot represents a biological replicate, highlighting the variance in transcriptomic profiles based on treatment and temporal progression. *n* = 3 biological replicates per condition. (C) Heatmap displaying the 1349 most variably expressed genes in MUG‐chor cells treated with DMSO, halofuginone or halofuginol across the indicated time points. The heatmap illustrates differential gene expression patterns and clustering of genes based on treatment and time course. *n* = 3 biological replicates per condition. (D) Gene Ontology (GO) analysis of differentially expressed genes in halofuginone‐treated cells over the full time course (0–72 h). The bar graph ranks the GO terms according to their significance, emphasizing the biological processes most impacted by the treatment. *n* = 3 biological replicates per condition. (E, F). Expression levels of DDIT3 and ATF4 as assessed by RNA‐seq analysis in MUG‐chor cells over 6 h, 24 h and 72 h following treatment with DMSO, halofuginone or halofuginol. The line plots represent the dynamic changes in expression levels of these stress response genes, indicating activation of stress pathways. Data represent mean ± standard deviation from three biological replicates per condition. (G) Quantitative PCR (qPCR) analysis of Integrated Stress Response (ISR) target genes (*ATF4*, *ASS1*, *DDIT3*) in MUG‐chor cells pretreated for 1 h with DMSO or ISRIB (500 nm), followed by halofuginone treatment (100 nm) for 6 h. ISRIB partially attenuated the transcriptional upregulation of stress response genes induced by halofuginone, indicating functional involvement of the ISR pathway. Data represent mean ± standard deviation from three biological replicates.

To elucidate further the mechanisms of action of halofuginone and halofuginol, we investigated early gene responses, prompted by the significant cell death observed by day three of treatment. Transcriptome analysis on MUG‐Chor cells was performed at multiple early time points (0, 6, 24, and 72 h) to capture the dynamic changes in gene expression. Time course analysis of gene expression revealed that the largest source of variance was the treatment duration, followed by the treatment type (Fig. 3A–C). This analysis highlighted distinct patterns of early and late gene activation. Differentially expressed genes throughout the time course were associated with several key metabolic pathways, including the aerobic electron transport chain, ATP synthesis, and processes regulating DNA damage and protein folding (Figs 3D and S3). Importantly, a significant upregulation of stress response genes *DDIT3/CHOP* and *ATF4* was observed, with both showing marked increases at the 6 h time point compared to the DMSO control (Fig. 3E,F). This upregulation persisted, but without significant differential expression between halofuginone and halofuginol, at the 24 and 72 h time points relative to the DMSO control (Fig. 3E,F). To further confirm the involvement of the ATF4‐DDIT3/CHOP axis in chordoma cell death, we have inhibited the ATF4 response pathway using ISRIB (integrated stress response inhibitor, a small molecule inhibitor that restores activity of the translation initiation factor eIF2B subunit beta (eIF2B) and counteracts the effects of eIF2α phosphorylation) [25]. When combined with halofuginone, ISRIB partially rescued cell viability (Fig. S4) as well as normalized the expression of *ATF4*‐dependent genes (Fig. 3G).

### 3.4 tRNA synthetase inhibition is associated with ATF4 stress response but not with TBXT modulation

**Fig. 4:**
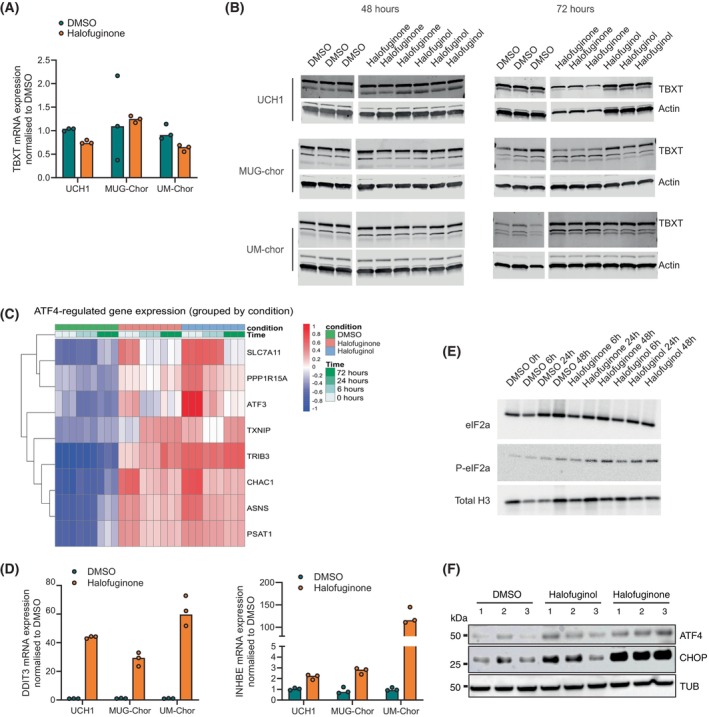
tRNA synthetase inhibition triggers ATF4‐mediated stress response without modulating TBXT expression. (A) qPCR analysis of TBXT mRNA expression in chordoma cell lines treated with halofuginone or DMSO for 48 h. TBXT mRNA expression remains unchanged. *n* = 3 biological replicates per condition. (B) Western blot analysis of TBXT protein expression in multiple chordoma cell lines following 48 h and 72 h of treatment with halofuginone or halofuginol. Beta‐actin is included as an endogenous loading control. Each condition is represented by three biological replicates. (C) Heatmap displaying the expression levels of ATF4‐responsive genes in MUG‐chor cells treated with DMSO, halofuginone or halofuginol across the indicated time points. The heatmap illustrates differential gene expression patterns and clustering of genes based on treatment and time course. (D) qPCR analysis of the ATF4 target genes, DDIT3 and INHBE, in chordoma cell lines treated with halofuginone or DMSO for 48 h. Expression of DDIT3 and INHBE is significantly induced in all cell lines. *n* = 3 biological replicates per condition. (E) Western blot analysis of key proteins in the p‐eIF2a‐ATF4 stress response pathway eIF2α and phospho‐eIF2α (p‐eIF2α), in MUG‐Chor cells treated with halofuginone or halofuginol at various time points 0, 6, 24, 48 h after treatment. Total histone H3 serves as an endogenous control. This panel highlights the activation of the stress response pathway over time. *n* = 1 biological replicates per condition. (F) Western blot analysis of ATF4 and CHOP in MUG‐Chor cells treated with DMSO, halofuginol, or halofuginone for 48 h. Each treatment condition includes three biological replicates (*n* = 3). Both ATF4 and CHOP, key effectors in the integrated stress response (ISR), show upregulated protein levels in response to halofuginol and halofuginone treatment, consistent with activation of the ATF4 pathway. Tubulin (TUB) is included as a loading control. *n* = 3 biological replicates per condition.

We then assessed whether these treatments altered the expression of TBXT, a gene previously reported to be responsive to other compounds [6, 9, 26]. TBXT expression remained unchanged in response to halofuginone or halofuginol 48 h after treatment, both at the gene expression and protein levels in multiple chordoma cell lines (Figs 4A,B and S5).

Instead, these compounds induced a pronounced ATF4 stress response, as evidenced by the upregulation of several *ATF4* target genes. RNA‐seq data show that several genes, including *Asparagine synthetase [glutamine‐hydrolysing] (ASNS)*, significantly increased at the 6 h time point, and the strength of the response was regulated in a gene‐specific manner over time (Fig. 4C). The induction of ATF4 targets such as *DDIT3* and Inhibin beta E chain (*INHBE*) was confirmed by qPCR on multiple cell lines at 48 h (Fig. 4D). Moreover, at 48 h after treatment we observed the activation of ATF‐eIF2α pathways with an increase in phosphorylated eIF2 (Fig. 4E), together with the induction of ATF4 and CHOP (Fig. 4F) at the protein level.

At the 72 h time point, the response to halofuginone or halofuginol was not accompanied by a consistent decrease in TBXT expression across cell lines (Figs 4B and S5); therefore, it supports the concept that the observed reduction in proliferation is not directly related to TBXT downregulation but is associated with the activation of an ATF4 stress response in multiple cell lines.

### 3.5 Halofuginone reduces chordoma tumour volume in an *in vivo* xenograft model

**Fig. 5:**
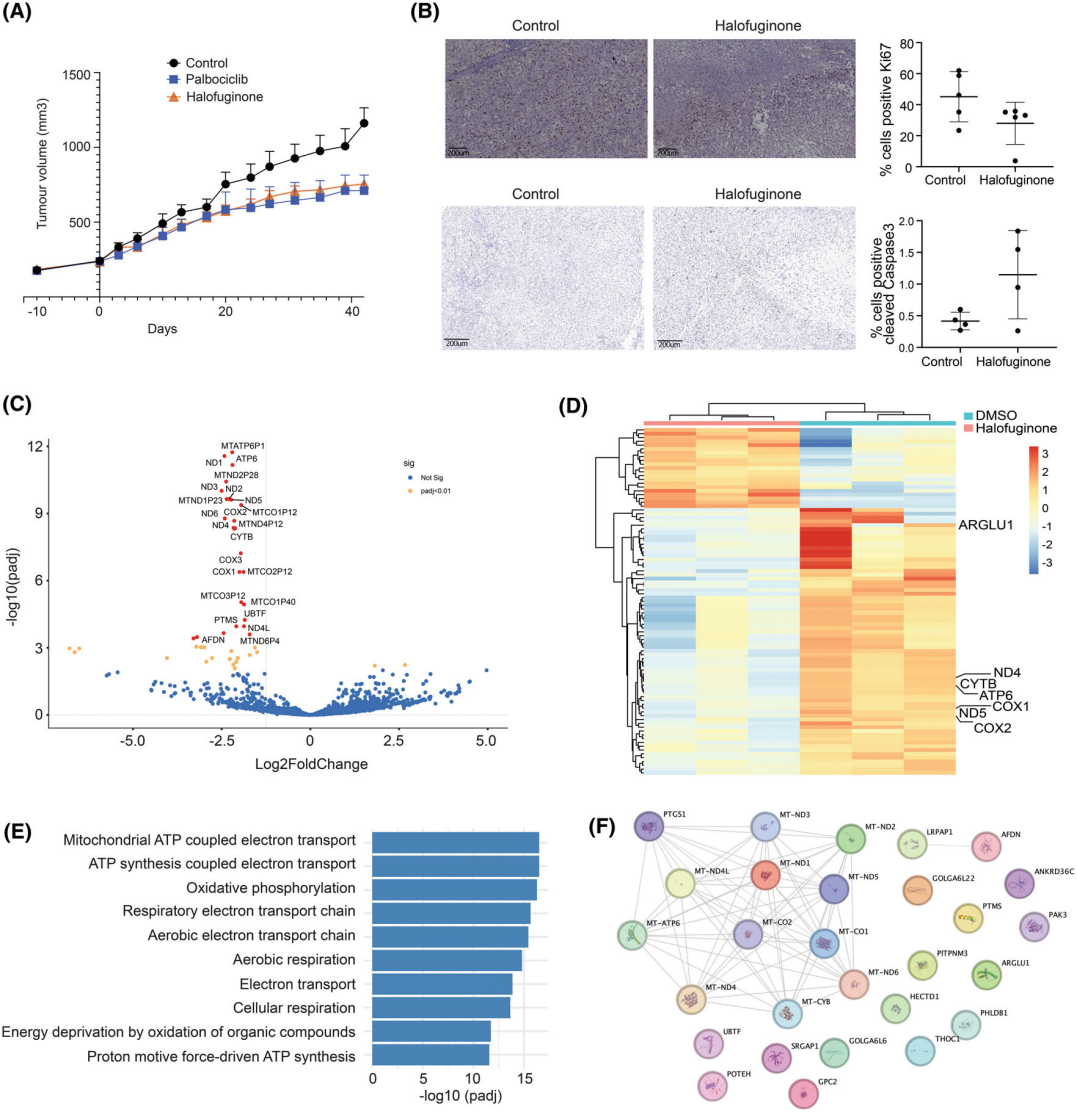
Halofuginone reduces chordoma tumour volume in an *in vivo* xenograft model. (A) Halofuginone treatment results in a significant reduction in tumour growth in the patient‐derived xenograft SF8894 mouse model, with effects comparable to the reference standard Palbociclib. Tumour volumes were measured over time, and data are presented as mean ± standard deviation for each treatment group. *n* = 5 animals per condition. (B) Representative immunohistochemistry images showing Ki‐67 staining (top, 10× magnification) and cleaved Caspase‐3 (bottom, 7× magnification) in tumour tissues from the SF8894 xenograft model after 40 days of treatment with either vehicle control or halofuginone. The right panels quantify the percentage of Ki‐67 positive cells (top), highlighting a reduction in proliferation in the halofuginone‐treated group, and cleaved Caspase‐3 (bottom) showing a higher percentage of dead cells in the halofuginone‐treated group. Scale bar = 200 μm. *n* = 5 (Ki‐67) or *n* = 4 (Caspase‐3) animals per condition. Data represent the mean ± standard deviation for each treatment group. (C) Volcano plot illustrating differential gene expression in tumour tissues from the SF8894 xenograft model following 40 days of halofuginone treatment. Genes with an adjusted *P* value (Padj) < 0.05 are highlighted in orange, genes with an adjusted *P* value (Padj) < 0.05 and Log_2_ Fold Change > 1.5 are shown in red, while non‐significant genes are shown in blue. Data are based on a minimum of four biological replicates. *n* = 3 animals per condition. (D) Heatmap depicting the most significantly altered genes in tumour tissues from the SF8894 xenograft model after halofuginone treatment. The heatmap illustrates distinct gene expression patterns in response to treatment. *n* = 3 animals per condition. (E) Gene Ontology (GO) analysis of differentially expressed genes in halofuginone‐treated tumour tissues, with the top GO terms ranked by significance. *n* = 3 animals per condition. (F) Protein–protein interaction network analysis of significantly altered genes in the halofuginone‐treated group, highlighting key pathways and interactions involved in the drug's mechanism of action. Each gene is shown in a randomly assigned individual colour. *n* = 5 animals per condition.

Finally, the anti‐tumour effects of the compounds were evaluated *in vivo* using the SF8894 PDX model of chordoma (Tables S6–S9). Halofuginone was tested alongside Palbociclib, a kinase inhibitor currently in clinical trials (Clinical Trial ref: NCT03110744), which served as a positive control. Both agents were generally well tolerated; however, halofuginone treatment resulted in a dose‐dependent weight loss of up to 15% at week 40 (Fig. S6). Both Palbociclib and halofuginone demonstrated statistically significant tumour growth inhibition (*P* < 0.05) in the SF8894 model (Fig. 5A), highlighting the therapeutic potential of halofuginone for chordoma.

Immunohistochemical analysis of treated tumours showed a reduction in proliferation marker protein Ki‐67 (Ki‐67) expression and an increase in cleaved Caspase‐3 expression, correlating with the observed decrease in tumour size (Fig. 5B). We also performed long‐read RNA sequencing to conduct full‐length transcript analysis on tumour samples from the PDX model treated with DMSO and halofuginone. However, due to sample degradation, reliable full‐length transcript analysis could not be achieved, and only gene‐level analysis was performed. Despite this limitation, the analysis revealed that most of the differentially regulated genes were downregulated in response to halofuginone treatment (Fig. 5C,D and Table S10). Gene Ontology and STRING analyses (Fig. 5E,F) identified an enrichment of genes associated with ATP‐dependent pathways, energy deprivation and significant regulation of mitochondrial respiratory‐related genes (Fig. 5E).

In conclusion, we have identified novel therapeutic candidates for chordoma, demonstrating both *in vitro* and *in vivo* efficacy. These compounds exert their effects through metabolic rewiring of chordoma cells, independent of TBXT.

## 4 Discussion

Our study identifies tRNA synthetase inhibitors, specifically halofuginone and halofuginol, as promising therapeutic agents for chordoma, a malignancy that currently lacks effective targeted treatments [6, 12, 27]. These compounds induced apoptosis in chordoma cell lines through mechanisms independent of TBXT regulation, instead triggering a pronounced ATF4‐mediated stress response. The induction of an ATF4 stress response, rather than modulation of TBXT, represents a significant departure from conventional approaches targeting chordoma [28, 29]. Our results align with previous studies highlighting the role of amino acid deprivation and subsequent activation of the integrated stress response in immune and cancer cells [22, 30, 31]. Notably, halofuginone and halofuginol treatment resulted in the upregulation of stress response genes such as *DDIT3*, *INHBE*, *ASNS* and *ATF4*, implicating these pathways in the observed apoptotic effects. Furthermore, co‐treatment with ISRIB—an ISR pathway modulator—partially attenuated the transcriptional response induced by halofuginone, suggesting that ISR activation contributes functionally to the cellular effects of the drug.

This ATF4‐mediated mechanism contrasts with earlier studies on histone deacetylase inhibitors and KDM demethylase inhibitors, which primarily focused on the suppression of TBXT expression [9]. While these earlier studies suggested that TBXT repression could be a viable strategy for chordoma treatment [27], our findings indicate that targeting metabolic stress pathways may offer an alternative and potentially more effective approach.

Our study also highlights the importance of early gene response analysis to understand the mechanisms underlying drug efficacy. Time course analysis revealed distinct early and late gene activation responses, with early upregulation of ATF4 and related stress response genes. This temporal gene expression pattern underscores the dynamic nature of the cellular response to tRNA synthetase inhibition and suggests that early intervention in these stress pathways may be critical for therapeutic success.

In our *in vitro* and *in vivo* model systems, halofuginone was effective at reducing chordoma cell viability, demonstrating its potential as a therapeutic agent for chordoma. In PDX models, halofuginone significantly inhibited tumour growth similarly to the cyclin‐dependent kinase 4/6 (CDK4/6) inhibitor Palbociclib, which is currently being tested in a clinical trial (Clinical Trial: NCT03110744). Determining whether patients with chordoma will benefit from halofuginone alone or in combination with other agents will likely require a clinical trial [32]. This is particularly noteworthy given that halofuginone has already shown promise in treating other conditions, such as multiple myeloma [22, 23] and SARS‐CoV‐2 infection [24], and has received orphan drug status for scleroderma [33].

## 5 Conclusions

In conclusion, our findings demonstrate that tRNA synthetase inhibitors, specifically halofuginone and halofuginol, induce apoptosis in chordoma cells through an ATF4‐mediated stress response, independent of TBXT regulation. This mechanism of action provides a new avenue for therapeutic development in chordoma, complementing existing strategies that target epigenetic modifications and transcriptional regulation. Further studies are warranted to optimize these compounds for clinical use and to explore their potential therapeutic benefit in combination with other treatments.

## Conflict of interest

A.P.C. and U.O. are cofounders of Caeruleus Genomics Ltd and are inventors on several patents related to sequencing technologies filed by Oxford University Innovations.

## Author contributions

APC and UO designed the study with contributions from LC, AMF and JCC. LC, JD, APC, EC, VG, FSA, GG, LL and GW acquired the data. APC also conducted data analysis. APC and LC generated the figures. APC and LC wrote the paper with input from all authors.

## Supporting information


**Fig. S1.** Halofuginone and halofuginol induce time‐ and dose‐dependent reductions in MUG‐Chor cell counts.

**Fig. S2.** Halofuginone induces cell death in chordoma cell lines.

**Fig. S3.** Gene ontology (GO) enrichment analysis of differentially expressed genes following halofuginone treatment at 6 h.

**Fig. S4.** ISRIB partially rescues halofuginone‐induced cytotoxicity.

**Fig. S5.** Quantification of TBXT protein expression in chordoma cell lines following treatment with halofuginone and halofuginol.

**Fig. S6.** Body weight monitoring during *in vivo* treatment with halofuginone and palbociclib.

**Table S1.** List of compounds targeting metabolic pathways and kinases used in this study.

**Table S2.** List of epigenetic compounds and novel epigenetic inhibitors used in this study.

**Table S3.** List of antibodies used in this study.
**Table S4.** List of primers used in this study.

**Table S5.** List of differentially expressed genes in RNA‐seq in MUG‐Chor cells treated with halofuginone for 3 days.

**Table S6.** Detailed outline of animal study.
**Table S7.** Detailed sample collection.
**Table S8.** Animal weight and agent tolerability.
**Table S9.** Agent efficacy and tumour volume data.

**Table S10.** List of differentially expressed genes in RNA‐seq in PDX animals treated halofuginone.

## Data Availability

The raw RNA sequencing data have been deposited in the GEO database under the accession number GSE275637. The analysis workflows for processing short‐read RNA‐seq data are available in the GitHub repository https://github.com/cribbslab/cribbslab, utilizing the pseudobulk pipeline. Long‐read RNA‐seq data were processed using the TallyTriN pipeline, accessible via https://github.com/cribbslab/TallyTriN. Subsequently, reads were mapped to features using the featureCounts workflow available in the cribbslab repository.
