# Supplementary figures and images for: ATF4‐mediated stress response as a therapeutic vulnerability in chordoma
Source: Mol Oncol. 2025 Nov 29;20(4):1008–21. doi: 10.1002/1878-0261.70176 (PMC13060635; doi:10.1002/1878-0261.70176)

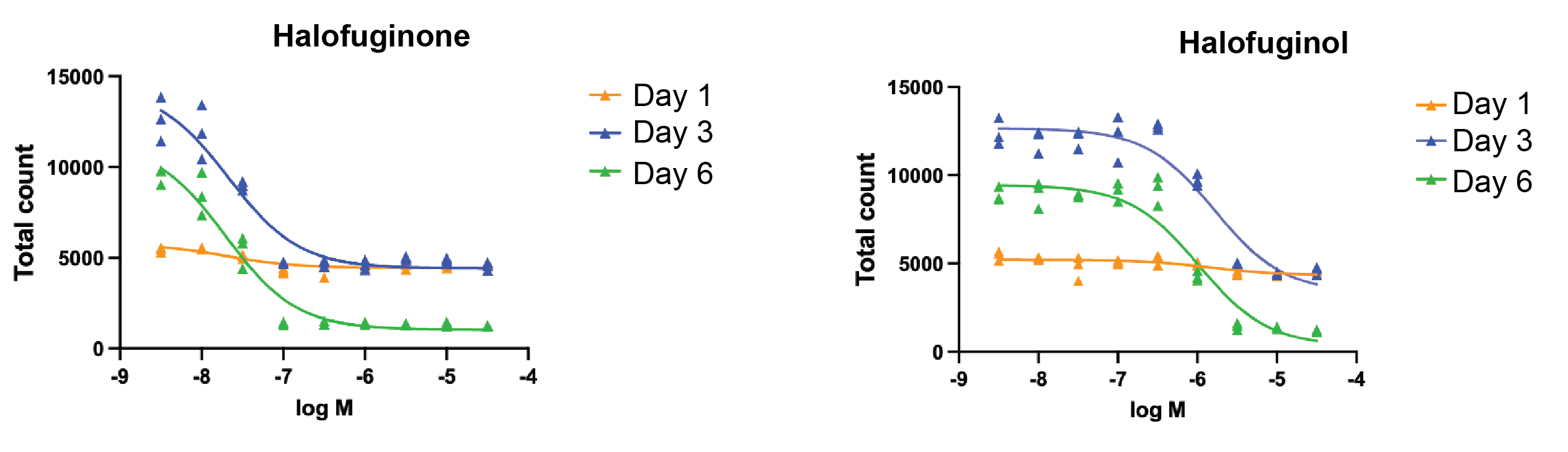

Supplement: Supplementary file 1 — Fig. S1. Halofuginone and halofuginol induce time‐ and dose‐dependent reductions in MUG‐Chor cell counts. [file MOL2-20-1008-s012.png]

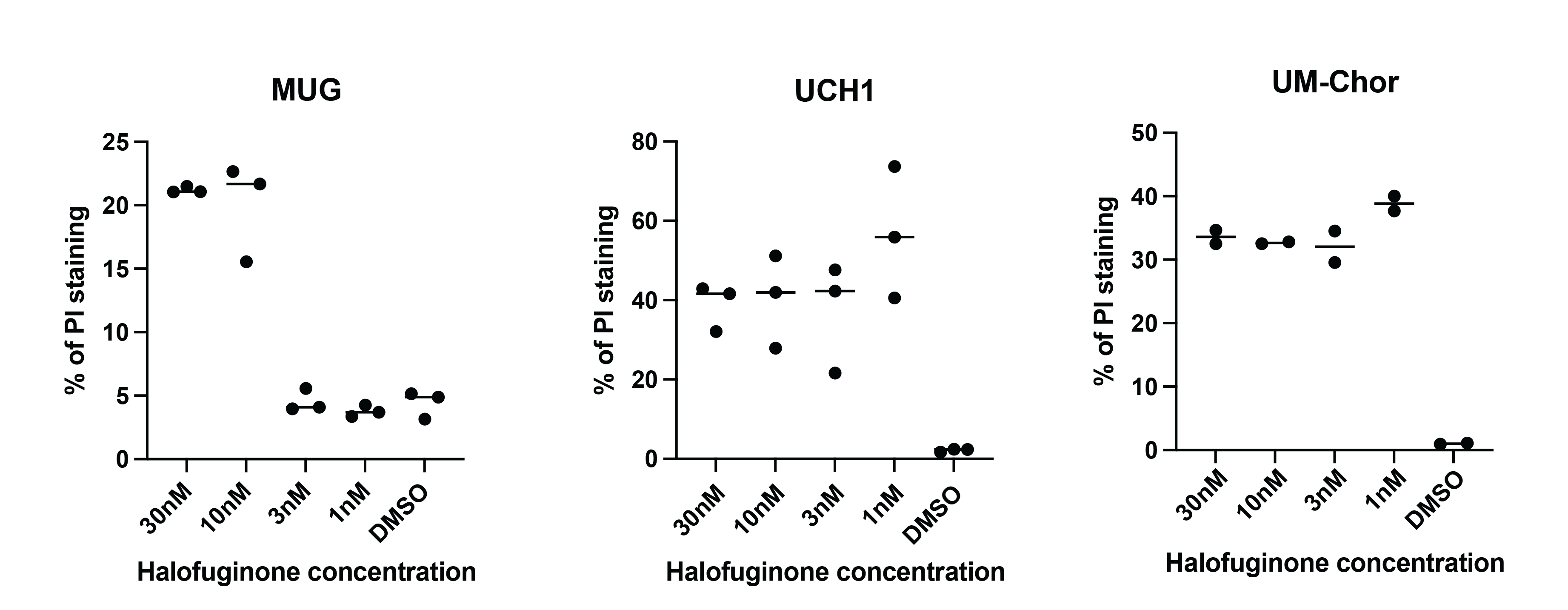

Supplement: Supplementary file 2 — Fig. S2. Halofuginone induces cell death in chordoma cell lines. [file MOL2-20-1008-s008.png]

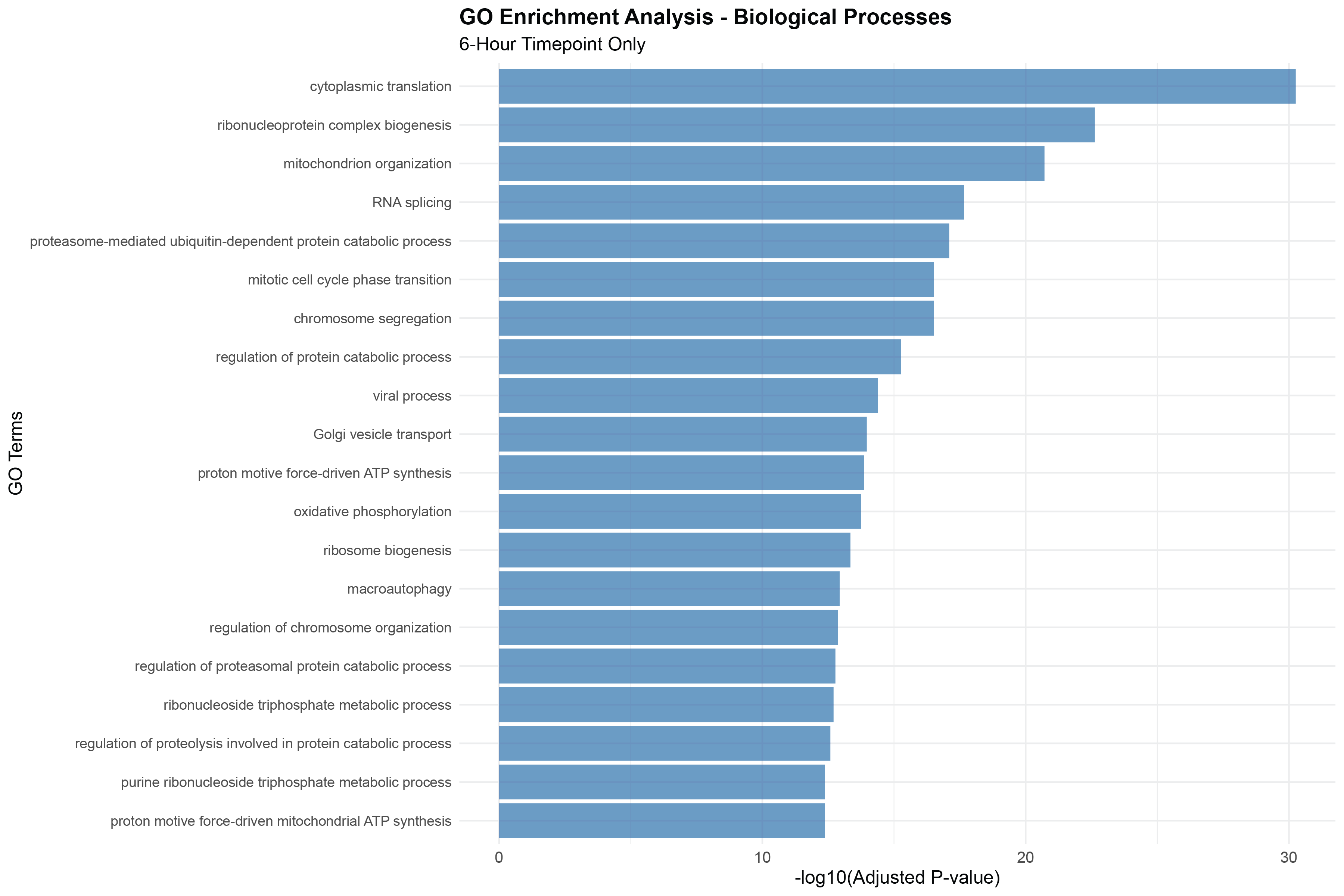

Supplement: Supplementary file 3 — Fig. S3. Gene ontology (GO) enrichment analysis of differentially expressed genes following halofuginone treatment at 6 h. [file MOL2-20-1008-s005.png]

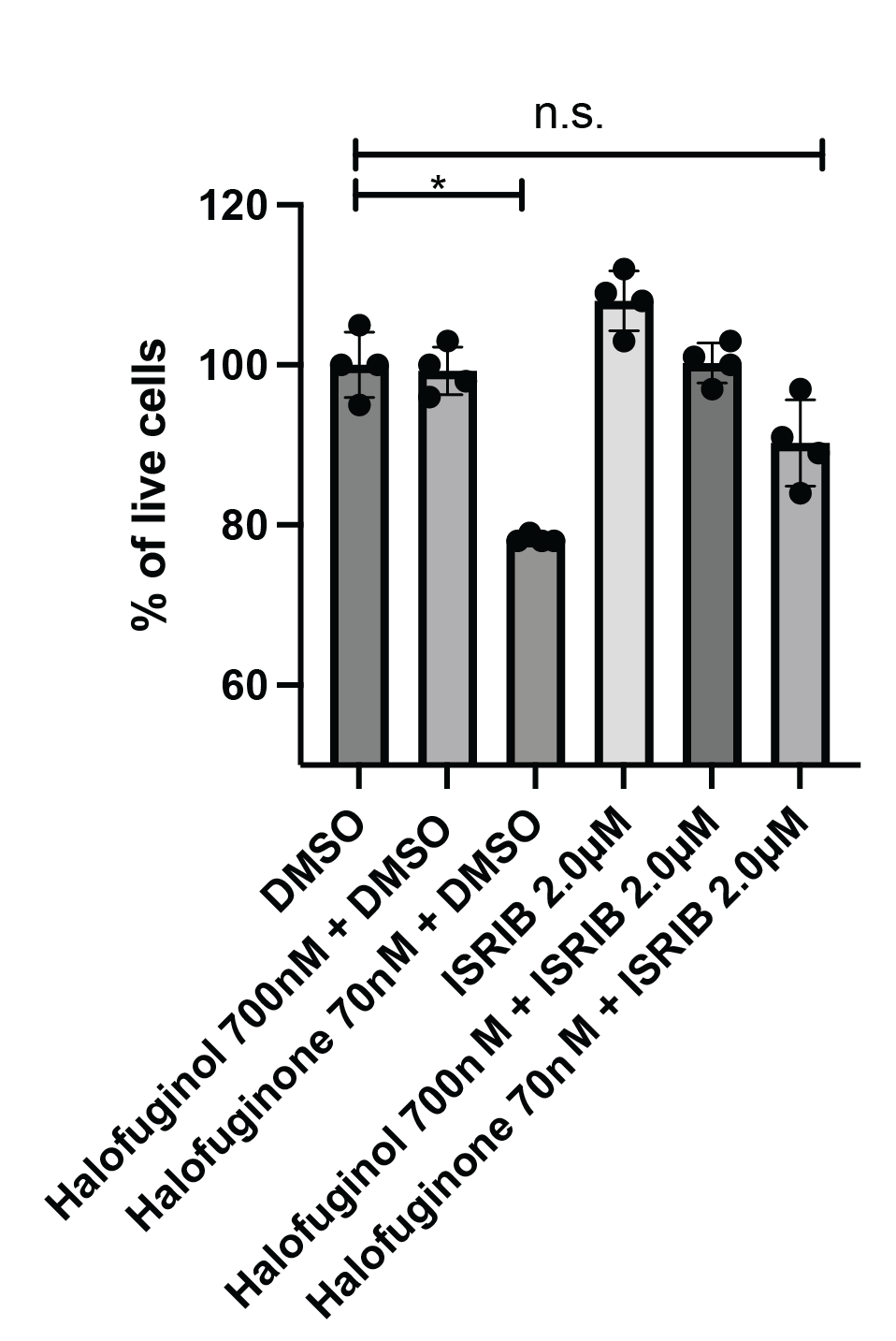

Supplement: Supplementary file 4 — Fig. S4. ISRIB partially rescues halofuginone‐induced cytotoxicity. [file MOL2-20-1008-s006.png]

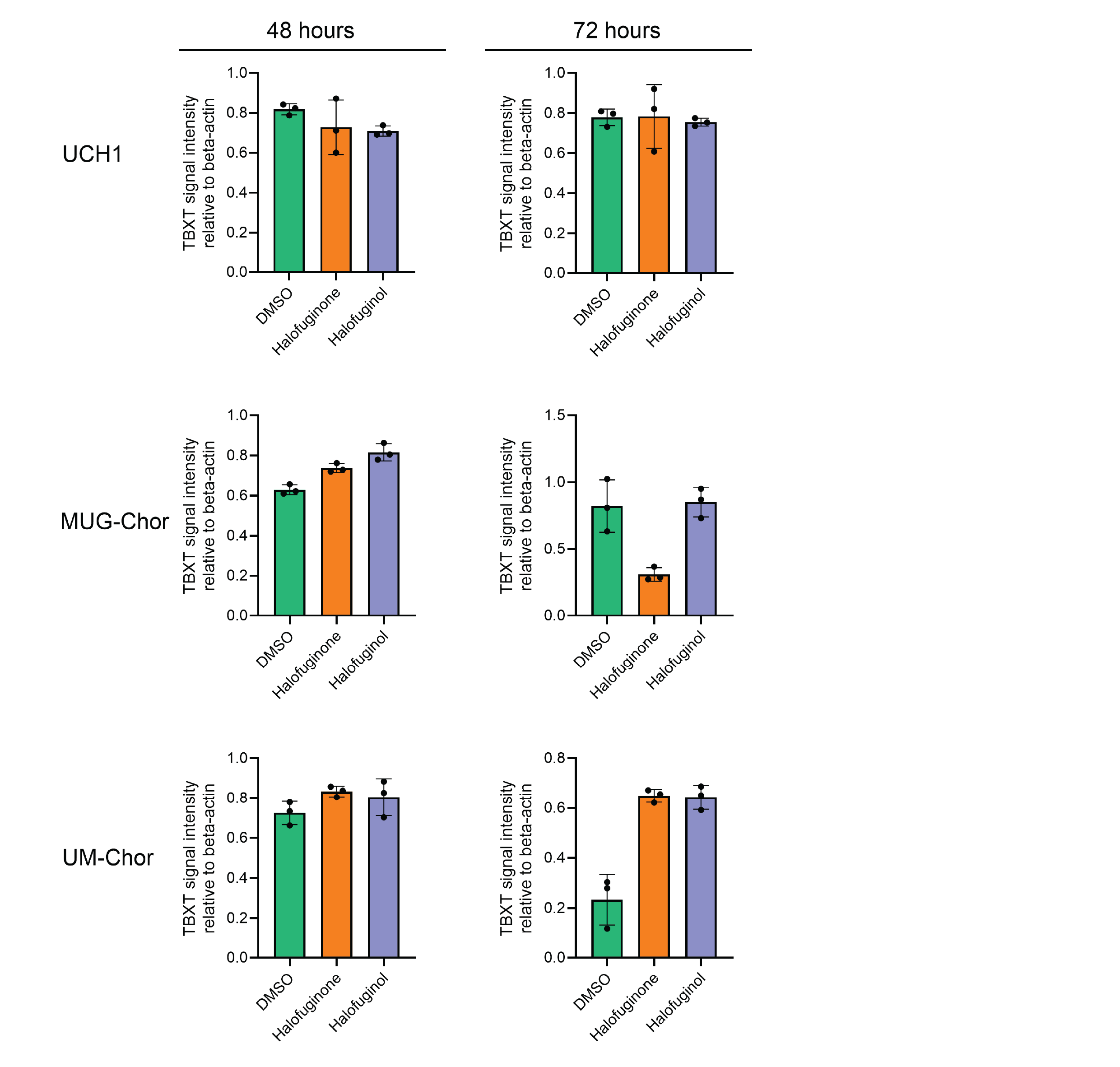

Supplement: Supplementary file 5 — Fig. S5. Quantification of TBXT protein expression in chordoma cell lines following treatment with halofuginone and halofuginol. [file MOL2-20-1008-s011.png]

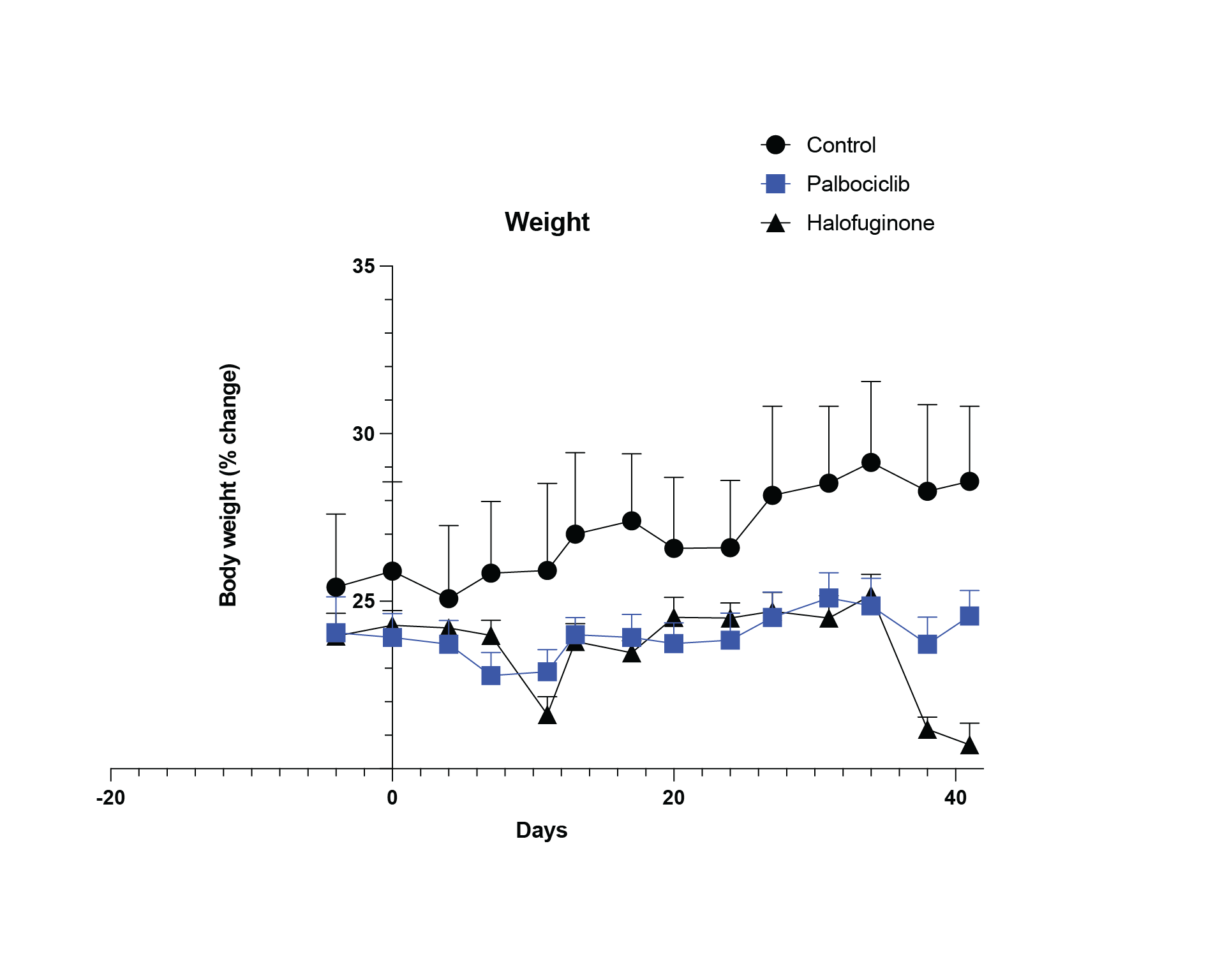

Supplement: Supplementary file 6 — Fig. S6. Body weight monitoring during in vivo treatment with halofuginone and palbociclib. [file MOL2-20-1008-s001.png]
