# Supplementary material for: ATF4‐mediated stress response as a therapeutic vulnerability in chordoma
Source: Mol Oncol. 2025 Nov 29;20(4):1008–21. doi: 10.1002/1878-0261.70176 (PMC13060635; doi:10.1002/1878-0261.70176)
Supplement: Supplementary file 9 — Table S3. List of antibodies used in this study. Table S4. List of primers used in this study. [file MOL2-20-1008-s004.docx]

**Supplementary Table S3. List of Antibodies used in this study.**

| **Protein** | **Brand** | **Cat. Number** |
| --- | --- | --- |
| Beta-Actin | Sigma | A5441 |
| TBXT (Brachyury A4) | Santa Cruz | Sc-374321 |
| PERK | Cell Signalling | cat#5683 |
| ATF4 | Cell Signalling | cat#11815 |
| eIF2-alpha | Abcam | cat #ab264253 |
| phospho-eIF2-alpha | Cell signalling | cat #ab32157 |
| Total H3 | Abcam | cat#ab1791 |

**Supplementary Table S4. List of primers used in this study.**

| Gene | Application | Fw Primer | Rev Primer |
| --- | --- | --- | --- |
| TBXT | qPCR | CCCGTCTCCTTCAGCAAAGTC | TGGATTCGAGGCTCATACTTATGC |
| DDIT3 | qPCR | ATGAACGGCTCAAGCAGGAA | GCAGATTCACCATTCGGTCAA |
