# Supplementary material for: ATF4‐mediated stress response as a therapeutic vulnerability in chordoma
Source: Mol Oncol. 2025 Nov 29;20(4):1008–21. doi: 10.1002/1878-0261.70176 (PMC13060635; doi:10.1002/1878-0261.70176)
Supplement: Supplementary file 11 — Table S6. Detailed outline of animal study. Table S7. Detailed sample collection. Table S8. Animal weight and agent tolerability. Table S9. Agent efficacy and tumour volume data. [file MOL2-20-1008-s003.doc]

Supplementary Table S6. Detailed Outline of Animal Study.

| **-N-**  **mice** | T_X_ | Dose  (mg/kg) | ROA / Schedule | Doses Administered | | Endpoint |
| --- | --- | --- | --- | --- | --- | --- |
|  |  |  |  | Total Dosed | T_X_ Day(s) |  |
| 5 | No Treatment | -- | -- | -- | -- | 42 |
| 5 | Palbociclib | 75 | PO / qd to end | 43 | 0-42 | 42 |
| 5 | Halofuginone | 1 | PO / qd to end | 43 | 0-42 | 42 |

Supplementary Table S7. Detailed Sample Collection.

| **Group** | **Tumor** | **# Samples Collected** | **Blood** | | |
| --- | --- | --- | --- | --- | --- |
|  |  |  | **Type** | **Time Point(s)** | **# Samples Collected** |
| No treatment | N | 5 | None | N/A | 0 |
|  | FFPE | 5 |  |  |  |
| Palbociclib | N | 5 | None | N/A | 0 |
|  | FFPE | 5 |  |  |  |
| Halofuginone | N | 5 | None | N/A | 0 |
|  | FFPE | 5 |  |  |  |
|  | | | | | |

Supplementary Table S8. Animal Weight and Agent Tolerability.

| Group | Weight Data (Day 42) | | Weight Nadir (Day 42) | | Drug Deaths | | | | | | |  |  |
| --- | --- | --- | --- | --- | --- | --- | --- | --- | --- | --- | --- | --- | --- |
|  | **Mean ± SD** | **%vD_0_** | **%vD_0(max)_** | **Day** | **D** | Day | **T** | Day | **B** | Day | **U** | | Day |
| No treatment | 29 ± 2 | +10% | -3% | 3 | **0** | -- | **0** | -- | **0** | -- | **0** | | -- |
| Palbociclib | 25 ± 2 | +3% | -5% | 6 | **0** | -- | **0** | -- | **0** | -- | **0** | | -- |
| Halofuginone | 21 ± 1 | -15% | -15% | 42 | **0** | -- | **0** | -- | **0** | -- | **0** | | -- |
|  | | | | | | | | | | | |  |  |

Abbreviations: %vD_0_= Weight change versus a study initiation (Day 0) measurement; %vD_0(max)_= Maximum weight loss versus a study initiation (Day 0) measurement; D= Death as a result of agent toxicity; T= Death as a result of technician error; B= Death as a result of tumor-related weight loss or cachexia; U= Cause of death cannot be determined

Supplementary Table S9. Agent Efficacy and Tumor Volume Data.

| Group | Mean ± SEM  (Day 42) | %TGI | p-value | Significant | PR (%TR) | CR | TFS |
| --- | --- | --- | --- | --- | --- | --- | --- |
| No treatment | 1161 ± 103 | -- | -- | -- | 0 | 0 | 0 |
| Palbociclib | 710 ± 105 | 49% | 0.0019 | Y | 0 | 0 | 0 |
| Halofuginone | 755 ± 20 | 44% | 0.0052 | Y | 0 | 0 | 0 |
|  | | | | | | | |

%TGI is defined as: Percent mean tumor growth at Day 42 versus Day 0 between treatment (TX) and untreated groups; Formula: %TGI = 1 – (TXf_avg_ – TXi_avg_) / (Cf_avg_ – Ci_avg_) where TX=mean tumor growth in drug treated animals; C=mean tumor growth in untreated animals; f=measurement on day 42; i= measurement on day 0. P-values <0.05 were considered statistically significant.
